# Enhancement of Flux Performance in PTFE Membranes for Direct Contact Membrane Distillation

**DOI:** 10.3390/polym12020345

**Published:** 2020-02-05

**Authors:** Ioannis N. Floros, Evangelos P. Kouvelos, Georgios I. Pilatos, Evangelos P. Hadjigeorgiou, Anastasios D. Gotzias, Evangelos P. Favvas, Andreas A. Sapalidis

**Affiliations:** 1 Institute of Nanoscience and Nanotechnology (INN), National Centre for Scientific Research (NCSR) “Demokritos”, 15310 Athens, Greece; i.floros@inn.demokritos.gr (I.N.F.); v.kouvelos@inn.demokritos.gr (E.P.K.); g.pilatos@inn.demokritos.gr (G.I.P.); a.gotzias@inn.demokritos.gr (A.D.G.); e.favvas@inn.demokritos.gr (E.P.F.); 2 Department of Materials Science & Engineering, University of Ioannina, 45110 Ioannina, Greece; hadjig@uoi.gr

**Keywords:** desalination, membrane distillation, hydrophobic-hydrophilic membrane, porous materials

## Abstract

This work focused on enhancing the flux on hydrophobic polymeric membranes aimed for direct contact membrane distillation desalination (DCMD) process without compromising salt rejection efficiency. Successful coating of commercial porous poly-tetrafluoroethylene membranes with poly(vinyl alcohol) (PVA) was achieved by solution dipping followed by a cross-linking step. The modified membranes were evaluated for their performance in DCMD, in terms of water flux and salt rejection. A series of different PVA concentration dipping solutions were used, and the results indicated that there was an optimum concentration after which the membranes became hydrophilic and unsuitable for use in membrane distillation. Best performing membranes were achieved under the specific experimental conditions, water flux 12.2 L·m^-2^·h^-1^ [LMH] with a salt rejection of 99.9%. Compared to the pristine membrane, the flux was enhanced by a factor of 2.7. The results seemed to indicate that introducing hydrophilic characteristics in a certain amount to a hydrophobic membrane could significantly enhance the membrane distillation (MD) performance without compromising salt rejection.

## 1. Introduction

One of the main issues that humanity is facing and is forecast to do in years to come is water availability. Water is an essential resource for the survival of living organisms; however, it is gradually becoming under threat in terms of quantity and quality. Membrane water treatment and, subsequently, desalination has the potential to provide a solution to both major issues. A number of countries due to geographical limitation and demographic changes have been driven to find new sources of freshwater, with most of them to have resorted to develop and employ desalination technologies, mainly reverse osmosis (RO).

A fairly new concept that steadily is gaining popularity in the field of desalination research is membrane distillation (MD), which, as the terms indicate, combines established desalination methods of membrane filtration and heating. In direct contact MD (DCMD), two streams exist; one hot usually at 50–80 °C, on the feed side (saltwater) of the membrane, and one cold, usually 18–25 °C, in the permeate side (sweep soft water), with a minimal constant differential pressure driving force [1]. Temperature induces vapor production on the hot side and, in turn, a vapor pressure driving force is established directed from the hot seawater side towards the cold soft waterside. This method aspires to be more competitive than established technology of RO or distillation, as the energy for pumping is significantly reduced compared to the RO process, and the energy for heating can be acquired more efficiently, compared to distillation, from renewable or waste heat resources, as low-grade heat can also be used [2,3,4,5]. Another important advantage of MD over RO is its potential of eliminating brine effluent streams and achieve zero liquid discharge (ZLD), as well as extract valuable minerals [6]. Recent attempts to improve the MD concept, by exploring the idea of making the membrane itself thermally active, have been carried out in order to increase process simplicity, productivity, and energy efficiency [7,8,9,10]. Another proposition is to use dense polymeric membranes, for which calculations have predicted a two-fold increase in MD vapor flux production by eliminating in parallel certain drawbacks found in conventional porous membranes [11]. These suggestions may prove important for the future direction, research, and development of the process. The MD process, therefore, may be particularly of interest to regions that have a reliable and adequate renewable heating source at proximity, such as a lot of sun hours or geothermal heat.

The four performance indicators that are important for this type of process are the steady-state water vapor flux, the purity of produced water, energy efficiency, and structural endurance. These indicators are either affected by membrane or process parameters. In order to optimize MD performance, the membrane parameters that need to be considered are (i) the thickness, pore size, and porosity to increase flux, the chosen average pore sizes lie in the range of 100–1000 nm [12,13,14,15,16], (ii) the wetting resistance, i.e., hydrophobicity of the pores to maintain purity of the product [17,18], (iii) the module design [19], the heat conduction of the material, membrane thickness, and porosity to increase energy efficiency [12,20], and (iv) thickness and porosity to increase structural endurance [21]. Comparatively less important is the latter indicator since the differential pressure applied in an MD process is low (usually <500 mbar, including hydraulic pressure), and, therefore, structural integrity is often sacrificed for the optimization of the remaining performance indicators [17]. More specifically, a thinner membrane favors a higher mass transfer, i.e., higher water vapor flux, but this also makes the membrane weaker structurally. A compromise must also be made as thickness affects thermal conductivity. A thicker membrane lowers thermal flux, i.e., reduces the effect of temperature polarization and, in turn, favors vapor water flux [22]. This can be addressed by focusing on developing composite porous membranes [20]. Hence, research and characterization of MD membranes should focus on the first three performance indicators, i.e., product flux, product purity, and energy efficiency.

Other than membrane parameters, MD performance indicators additionally depend on parameters related to the process itself, including feed streams’ compositions, temperature difference, and fluid velocities [22,23] and, consequently, Reynolds numbers as well as hydraulic pressure [24]. Feed concentrations are usually difficult to adjust, while the last two parameters can be altered, keeping in mind that these are the main parameters that affect the energy efficiency of the process.

From previous research [17], using a hydrophobic membrane (i.e., water contact angle (WCA) > 90°) has proven to satisfy the four aforementioned key performance indicators. Widely used polymers in an MD application are polyvinylidene fluoride (PVDF), polypropylene (PP), and polytetrafluoroethylene (PTFE), all of which form hydrophobic porous membranes. A large number of studies have focused on making super-hydrophobic surfaces in order to increase wetting and scaling resistance [25]. While others have looked into applying hydrophilic characteristics to hydrophobic porous membranes or vice versa [26,27,28], which has the effect of drawing water molecules in the membrane pores. Although this tends to increase the degree of temperature polarization, which, in turn, theoretically, would have a negative effect on flux. Interestingly, it has been observed that the opposite effect takes place, and flux increases [29]. It is suggested that this happens because by partly filling the pores with water, the flux of pore vapor travels a shorter distance. In other words, the limiting factor of flux is not temperature polarization but the length of vapor transport within the pores. In order to modify the surface of membranes and introduce hydrophilic or hydrophobic characteristics, a number of methods have been applied, both chemical and physical, including, among others, dip coating, grafting, etching, irradiation, and plasma [30,31,32].

PTFE was chosen as the polymeric membrane material as it is comparatively more hydrophobic, which increases wetting resistance, as well as it has a very good chemical resistance profile [30]. In this regard, this work focused on producing a novel PTFE-PVA (polytetrafluoroethylene-polyvinyl alcohol) hydrophobic membrane with hydrophilic moieties to enhance water permeability while maintaining desirable salt rejection levels. Recently, successful hydrophilic coating of PTFE has been undertaken by Zhao et al. 2019 [33], Li et al. 2019 [34], and Villabos-Garcia et al. 2018 [35], but also in the past by Song et al. 2017 [36], Park et al. 2015 [37], Mansouri et al. 1999 [38], and Prasad et al. 1987 [39], focusing, however, on different membrane applications. Although PTFE is a difficult polymer to work with and has little compatibility with other polymers, PVA from previous studies has worked satisfactorily with PTFE electrospun membranes [40,41,42,43,44].

This work explored the produced membranes’ performance and characteristics. The part of membrane performance looked into MD evaluation, while the part of membrane characteristics examined the effectiveness of PVA deposition both qualitatively and quantitatively by means of WCA, pore size calculations, porosity, liquid entry pressure (LEP), and bubble point pressure (BubP).

## 2. Materials and Methods

### 2.1. Materials

PTFE flat porous disc-shaped membranes laminated with polypropylene (PP) (the PTFE side is used as the feed side, while the PP or support side is used as the permeate side), with pore diameter (*d*_pores_) of 220 nm and membrane diameter (*d*_membrane_) of 47mm, were purchased from *Filtres-Fioroni*, while PVA granules (*M*w = 205,000 g/mol, 88 mol% hydrolysis), isopropyl alcohol (IPA, 99%), ethanol pure (EtOH, 99.9%), citric acid (CA) granules, and glutaraldehyde (GA, 25% aqueous solution) were purchased from Sigma-Aldrich (Athens, Greece). Deionized water (conductivity < 20μS/cm) was used in all aqueous solutions (aq.sol.).

### 2.2. Membrane Coating Procedure

**Figure 1:**
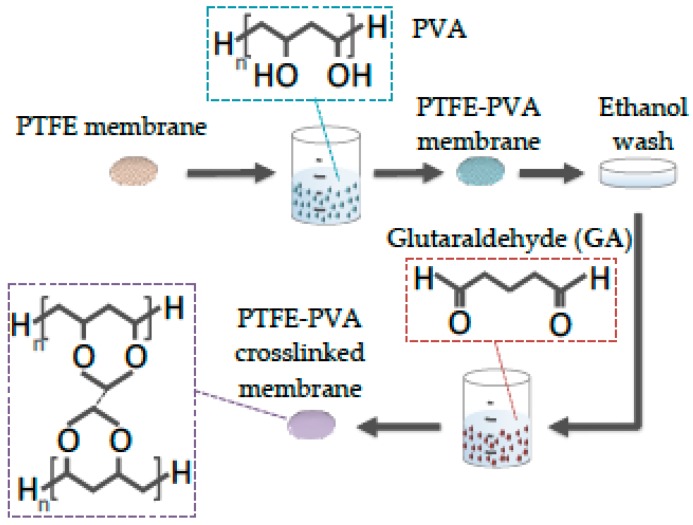
Step-by-step depiction of polyvinyl alcohol (PVA) deposition and cross-linking on polytetrafluoroethylene (PTFE) membrane.

In this work, the modification procedure, shown in Figure 1, was followed; PTFE hydrophobic membranes were submerged in various concentrations of PVA aqueous solutions (0.05, 0.1, 0.5, and 1.0 wt.%) of approximately 100 mL for 24 h. Subsequently, the membranes were washed briefly with a PVA non-solvent, EtOH, so as to enhance the PVA impregnation of the surface-pore area of the membrane. Following this step, the membranes were immersed directly into the PVA cross-linking solution (0.125 g/mL GA and 0.02 g/mL of CA in water) for 24 h. This time duration for the impregnation as well as the cross-linking was chosen since adsorption kinetics are expected to be slow between the hydrophilic polymer and the hydrophobic porous membrane, and since various polymer solution concentrations were used, the long submersion time was considered necessary to eliminate any deviations due to diffusion limitations. Afterward, the modified membranes were washed for 3 h at 60 °C with pure water to remove any uncross-linked polymer and remaining reagents. Finally, they were dried in an oven at 40 °C and stored dry in a sealed container at room conditions. The membranes were labeled based on the dipping PVA solution that they were immersed in, i.e., PTFE for the pristine membrane, PTFE-0.05 for the dipping PVA solution of 0.05 g/100 mL, and so on.

### 2.3. Membrane Characterization

The surface of membrane samples was subjected to FT-IR in order to deduce the chemical consistency and hence whether the method followed for PVA deposition was successful. The transmittance spectra of the film samples were measured on a Nicolet Is50 instrument (ATR Smart ITX Diamond, Madison, WI, USA).

The membrane surface was also probed using SEM in order to obtain an insight into surface morphology and patterns. The microscope used was JEOL JSM 7401F (Tokyo, Japan) Field Emission Scanning Electron Microscope. The samples prior to viewing, in order to increase specimen conductivity and thus image clarity, were given platinum sputtered coating and attached to the microscope’s holder with carbon tape.

One of the most important membrane parameters in the MD process is liquid entry pressure (LEP). This value is desired to be as high as possible since a high value will mitigate pore wetting. Pore wetting is an undesired effect seen in the MD process as this allows for the degradation of purity of produced water. It follows that the trans-membrane pressure needs to be lower than the LEP value. Calculating LEP offers an insight into the pore dimensions, pore size, and geometry, as well as hydrophobicity of the membrane’s surface area. This is reflected in the basic model used to calculate LEP, the hydraulic pressure difference between feed, *P_F_*, and permeate, *P_P_*, comprising of a single equation, which relates the value of LEP (Pa) with the surface tension of the fluid *γ* (N m^−1^), the maximum pore radius *d_max_* (m), the fluid contact angle *θ* (°) and assumes an intrinsic parameter for the pores’ geometry *B* (-). The simplest assumption is for the pores to have cylindrical geometry; this is known as the Franken equation [45]. In this case, the parameter *B* is equal to one, and the equation has the form of

(1) $$LEP=\Delta P=P_{F}-P_{P}=-2\times \gamma \times \frac{cos\theta }{d_{max}/2}$$

The above relationship is equivalent to a Young–Laplace equation. More recent efforts have focused on improving the accuracy of Franken’s equation by introducing new variables and equations [46,47].

Bubble point pressure was calculated using the procedure described in ASTM F316–03 [48]. The membranes were fitted in a holder having been beforehand wetted with the selected liquid, which is assumed to be able to defuse within the pores. In this respect, IPA was chosen (*γ* = 23 mN/m). A nitrogen stream was constantly directed to the feed side of the membrane using a back-pressure regulator in order to maintain the desired pressure. The permeate side of the membrane was covered at a height of about 1 cm with IPA. The N_2_ pressure was gradually increased until the first constant rate of bubble flow was observed coming from the center of the membrane, at the permeate side. This is the point at which the bubble point pressure was noted.

The equation used to calculate the max pore diameter (*d_max_*) (m) using the bubble point pressure (*P_BubP_*) (Pa) is given below [48].

(2) $$d_{max}=\frac{C\times \gamma }{P_{BubP}}$$

where *C* is a constant assumed to be equal to one, and *γ* (N/m) is the surface tension of the chosen fluid.

The porosity of membranes was measured through the gravimetric method. The pores were filled with a liquid, pure IPA. The porosity *ε* (%) was calculated by subtracting the weight of pre-IPA (*w*_1_) from the post-IPA membrane (*w*_2_), as well as accounting for membrane thickness *t_m_* (m) and surface area *A_m_* (m^2^) and IPA density *d_IPA_* (kg/m^3^).

(3) $$\varepsilon =\frac{w_{2}-w_{1}}{A_{m}\times t_{m}\times d_{IPA}}$$

**Figure 2:**
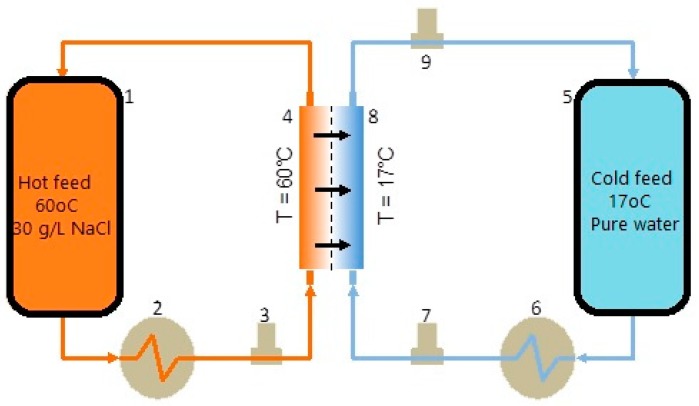
Experimental membrane distillation (MD) set-up of hot feed (30 g/L salt and at 60 °C) and cold feed (pure water and at 17 °C). 1-hot feed water tank, 2-hot feed heat exchanger, 3-mass flow controller of hot stream membrane inflow, 4-membrane side of hot feed, 5-cold feed water tank, 6-cold feed heat exchanger, 7-mass flow controller of cold stream membrane inflow, 8-membrane side of cold feed, 9-mass flow controller of cold stream membrane outflow.

Calculations of membrane MD performance were carried out using a custom laboratory unit (Figure 2). The hot water feed, ${\dot{v}}_{Hin}$ (mL/min), with a NaCl concentration of 30 g/L and a conductivity of 46.7 mS/cm, was pumped at a fixed rate of 40 mL/min and a constant temperature of 60 °C. The cold feed of pure water, ${\dot{v}}_{Cin}$ (mL/min), with a conductivity of 7 μS/cm, was pumped similarly at a fixed rate of 40 mL/min and a constant temperature of 17 °C.

The flow rates of both feeds were controlled by an electronic pump drive, while saltwater feed, softwater membrane inlet, and outflow were recorded electronically using *Bronkhorst CORI-FLOW™ series* mass flow meters. The data were continuously logged on a pc. The temperature of both feeds was measured upstream from the membrane with a handheld temperature probe, *Ω-Omega*. The conductivity of samples, collected periodically, was measured by a *Consort C1010* bench-scale conductivity meter.

Membrane water flux, *J_W_* (L·m^−2^·h^−1^), was calculated based on measurements made for a volumetric flow rate of vapor permeate, ${\dot{v}}_{Perm}$ (L/h), the effective membrane area, *A_Eff_* (m^2^), which was 7 × 10^−4^ m^2^, and for time, *t* (h).

(4) $$J_{W}=\frac{{\dot{v}}_{Perm}}{A_{Eff}}$$

where permeate flux was calculated by subtracting volumetric flow of cold feed, ${\dot{v}}_{Cin}$ (L/h), with that of the cold outlet, ${\dot{v}}_{Cout}$ (L/h).

(5) $${\dot{v}}_{Perm}={\dot{v}}_{Cout}-{\dot{v}}_{Cin}$$

Membrane salt rejection, *R* (*%*), was estimated by calculating salt concentration in hot feed *C_Hin_* (30 g/L) and the permeate *C_Perm_* (g/L)

(6) $$R=\frac{C_{Hin}-C_{Perm}}{C_{Hin}}\times 100$$

where *C_Perm_* is a function of the relationship below based on conductivity measurements of the cold outlet, *σ_Cout_* (mS/cm), and hot feed, *σ_Hin_* (mS/cm).

(7) $$C_{Perm}=f\left(\frac{{\dot{v}}_{Cout}\times \sigma_{Cout}-{\dot{v}}_{Cout}\times \sigma_{Cin}}{{\dot{v}}_{Cout}-{\dot{v}}_{Cin}}\right)$$

## 3. Results and Discussion

### 3.1. FT-IR

**Figure 3:**
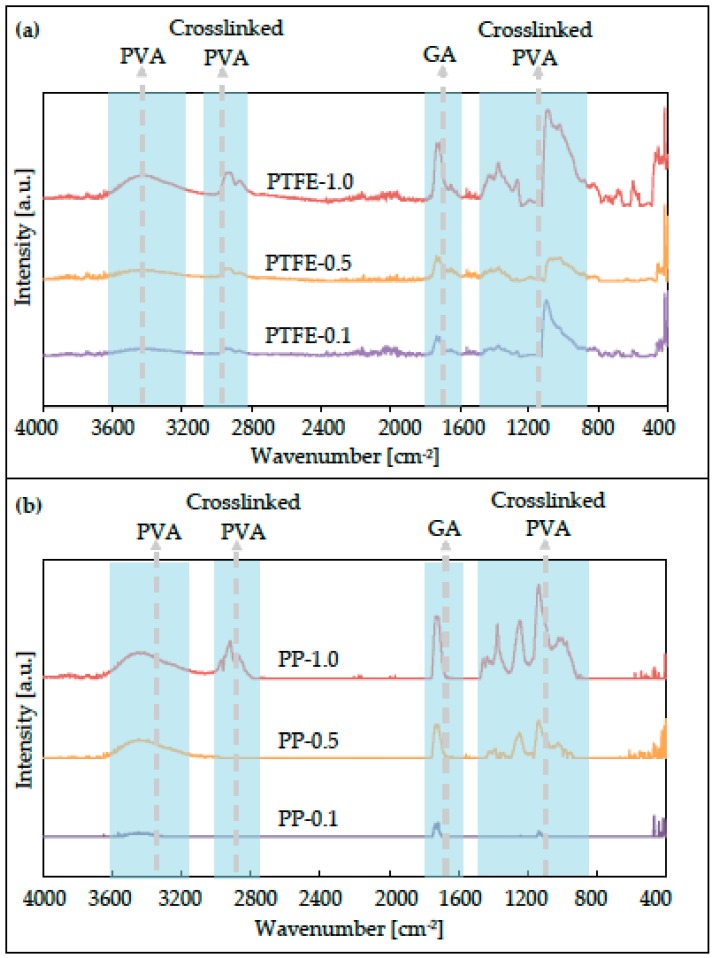
Spectra of PVA-coated feed side (PTFE) (**a**) and permeate side (PP) (**b**) subtracted from pristine spectra of untreated PTFE and polypropylene (PP) surfaces of the membrane, respectively. Highlighted with blue are the bands that represent pertinent to this work’s modification chemical bond groups of PVA, glutaraldehyde (GA), and cross-linked PVA.

From the characterization techniques employed, it was clear that PVA was successfully deposited on the PTFE membrane surface. The FT-IR plots clearly showed the bands representative of chemical groups both found in PVA and GA (Figure 3). The same behavioural traits could be observed in both the IR spectrums of feed (PTFE) and permeate (PP) membrane sides. Qualitatively, there was a clear relationship between PVA deposition and the corresponding peaks, i.e., as PVA deposition increased, so did the intensity of the peaks. Although peaks that were associated with GA did appear in the spectrum, PVA was the one for which concentration was increased while that of GA in all cases remained fixed.

A number of peaks could be identified from the plots. For all modified membranes, common wavelengths could be identified (a) in the band 3500–3000 cm^−1^, (b) in the band of 3000–2800 cm^−1^, (c) at the peak of 1720 cm^−1^, (d) in the band of 1380–1370 cm^−1^, (e) in the band of 1100–950 cm^−1^.

(a) In the region of 3500–3000 cm^−1^, there existed a broad peak that was linked to hydroxyl groups (–OH), a characteristic of the PVA polymer.
(b) In the region of 3000–2850 cm^−1^, there existed two sharp peaks at 2930 cm^−1^ and 2860 cm^−1^, indicating the existence of alkali groups (C–H). The last peak is relevant to aldehyde groups [49].
(c) The peak at a wavelength of 1720 cm^−1^ was associated with carbonyl groups (C=O) and, more specifically, to aldehyde bonds. These groups were characteristic of the cross-linker GA. This showed that the GA was present in excess in the solution, which, in combination with the chosen long reaction period of 24 h, allowed for partially unreacted molecules to get entangled within the cross-linked PVA framework [50].
(d) The peaks observed in the band of 1380–1370 cm^−1^ indicated the presence of alkane and aldehyde groups, which were a result of deposition and cross-linking of PVA with GA.
(e) The peaks appearing in the band of 1250–950 cm^−1^ were representative of ether bonds (C–O–C), which, in this case, belonged to dioxane formed by cross-linked PVA.

Additionally, peaks appearing in the band of 1450–1420 cm^−1^ and 1270–1240 cm^−1^ could be attributed to groups of alkane (CH_2_ or CH_3_), hydroxyls (–OH), and ethers (–CO).

### 3.2. SEM

**Figure 4:**
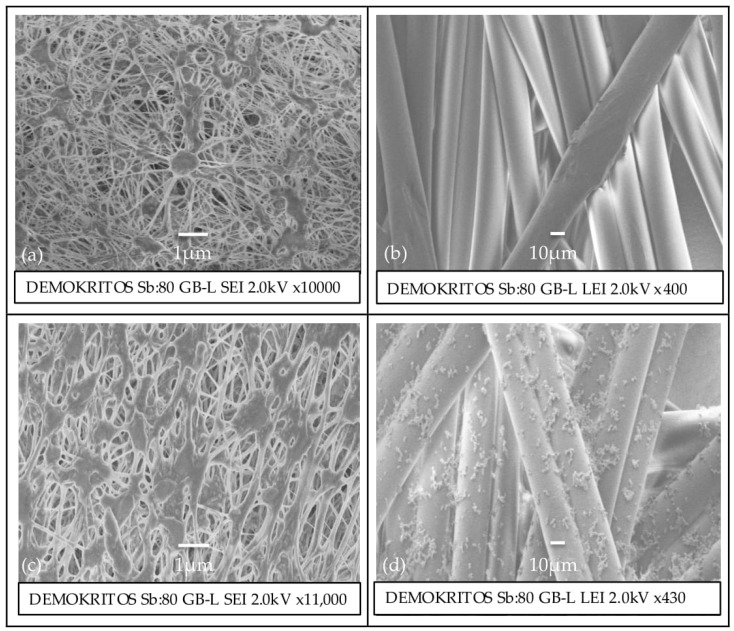
SEM images of pristine membrane surface of the feed side (**a**) and permeate side (**b**); SEM images of PTFE-1.0 surface of feed side (**c**) and permeate side (**d**).

From the SEM images (Figure 4d), the first observation was the long tubular fibers, about 15 μm thick, characteristic of non-woven membrane support. The second observation was that these fibers were partly covered with relatively smaller grain-like granules that had randomly formed pockets of agglomerates. Getting closer became apparent that these granules had a spherical shape and a size ranging from 1.1–1.4 μm. These were assumed to be PVA granules that had formed agglomerations due to hydrophilic interactions. Similar structures were not observed when the PTFE side was probed, indicating less PVA adhesion, as expected (Figure 4c).

### 3.3. Water Contact Angle and Porosity

Another indirect but reliable method to check for PVA deposition was to measure the WCA of the membrane surface. This could offer an insight into the degree of hydrophilicity of the surface of the modified membranes (see Table 1), which was influenced by two key membrane parameters, (i) the geometric morphology/roughness at the nano-scale and (ii) the surface energy. PTFE polymer consisted of chains of fluoride groups (–CF_2_), which decreased the surface energy (<20 mN/m) and thereby induced hydrophobic characteristics. To a lesser extent, chemical groups in PP also produced a low surface energy profile (30 mN/m). In contrast, PVA, as well as its cross-linked derivative, partly consisted of groups, such as hydroxyls and ethers, that increased surface energy and, as such, the hydrophilic nature of the surface.

Evidently, there was a correlation between PVA concentration in the dipping solution and the water wetting of the produced membranes. The decrease in the WCA reached 90% for PTFE-1.0 compared to the original PTFE. It was interesting to note that when both sides of the membrane were tested for WCA, there was a significant deviation in the rate of WCA reduction with the support side became comparatively more hydrophilic than the feed side. As expected, the increase of PVA solution concentration decreased the porosity of the final membrane. This was attributed to the increased penetration of polyvinyl alcohol chains into the porous matrix due to the higher concentration gradient. This could also be partly corroborated by the PVA deposition seen on the SEM images (Figure 4).

### 3.4. Bubble Point Pressure and Liquid Entry Pressure

The common trend observed by using both methods was the decreasing pore size calculation for increasing PVA dipping solution concentration, which followed a similar decreasing trend as porosity and WCA. The added advantage of the BubP method was that it could be used for both hydrophobic and hydrophilic porous surfaces, while LEP-Franken’s model was limited to hydrophobic porous surfaces. In recent years, the model has been evolved to address this limitation using more sophisticated computational methods and considering additional parameters. Therefore, the evolved model is able to give a more reliable prediction for less hydrophobic porous materials [47].

Similarly to WCA, the LEP results revealed a close relation between PVA concentration deposition and observed properties (Table 2). From the relevant experimental data, there was a clear relationship between PVA solution concentration and BubP until sample 0.1. In increasing concentrations, there was no clear relation, and this could be possibly attributed to pore geometry alteration.

### 3.5. Desalination Performance Evaluation: Membrane Distillation Flux and Salt Rejection

When tested for their DCMD performance, the hydrophobically modified membranes exhibited satisfactory performance, both in terms of flux and salt rejection. Membranes coated with a PVA solution of 0.05% and 0.10% had a flux of 8.8 and 12.2 LMH, respectively, while having a salt rejection consistently above 99.9%.

Therefore, the optimum membrane in terms of flux and salt rejection was found to be PFTE-0.10 since it had significantly higher flux and a high salt rejection of 99.9%. The salt rejection translates to a salt concentration of well below the upper limit of 600 ppm [51], which is identified as the upper limit for good quality drinkable water. It is worth noting that freshwater is considered water that has a salt concentration of less than 1000 ppm (USGS, EPA) [51]. As could be seen from Table 3, the relationship between flux and salt rejection was almost unchanged for increasing concentration of PVA coating.

The performance of the developed membranes could be compared to that of previous works, as shown in Table 4.

## 4. Conclusions

Hydrophilic characteristics were successfully introduced to porous PTFE membranes aimed for direct contact membrane distillation desalination. A negligible amount of PVA was introduced to the membranes by solution dipping followed by a cross-linking step, using glutaraldehyde. To investigate the effect of the dipping solution, a series of PVA concentrations up to 1 wt.% in H_2_O was used. The success of membrane modification was tested using standard characterization techniques, including WCA, LEP, BubP, porosity, FT-IR, and SEM. From the SEM images, it was observed that PVA chains formed on the membrane supported fibers’ grain-like structures of the order of 1 μm. Moreover, the modified membranes were used in a laboratory-scale unit where they were tested for their MD performance in terms of product flux and purity. The characterization results showed that due to the PVA coating, the modified membranes had reduced hydrophobicity, lower LEP, BubP, and porosity, indicating the penetration of the PVA chains into the porous network of the membranes. Desalination evaluation of the modified membranes showed a remarkable increase (2.7 times fold) in water flux while retaining a high salt rejection efficiency of 99.9%. Finally, this work showed that the hydrophobic PTFE membrane, although chemically inert, could easily undergo modification using hydrophilic PVA, via dip-coating solution, and remain intact under MD desalination conditions.

## Figures and Tables

**Table 1:** Results of water contact angle (WCA) for feed side (PTFE, polytetrafluoroethylene) and permeate side (PP, polypropylene) membrane surfaces as well as the calculated membrane porosity.

Membrane	WCA_PTFE_ (°)	WCA_PP_ (°)	Porosity (%)
PTFE	128 ± 2	122 ± 3	69.3
PTFE-0.05	115 ± 3	110 ± 2	67.8
PTFE-0.10	95 ± 2	86 ± 3	66.4
PTFE-0.50	56 ± 2	43 ± 2	62.6
PTFE-1.00	<5	<5	57.2

**Table 2:** Results of bubble point pressure (BubP) and liquid entry pressure (LEP) and maximum pore size calculation (*d_max_*) using Franken’s equation γ_H2O_ = 0.072 Ν/m, Β = 1.

Membrane	BubP (bar)	*d*_max-BubP_ (nm)	LEP (bar)	*d*_max-LEP_ (nm)
PTFE	1.2	160	3.92	449
PTFE-0.05	1.5	128	3.90	312
PTFE-0.10	1.9	101	3.60	97
PTFE-0.50	1.9	101	<0.2	N.D.
PTFE-1.00	1.5	127	<0.1	N.D.

N.D.—cannot be determined.

**Table 3:** Membrane distillation performance–water flux vs. salt rejection.

Membrane	Flux LMH	Salt Rejection (%)
PTFE	4.5	99.9
PTFE-0.05	8.4	99.9
PTFE-0.10	12.2	99.9

**Table 4:** Comparison of this work to previous research. * PET—Polyethylene terephthalate, ** CS-PEO—chitosan-polyethylene oxide.

Source	Type	LEP	Flux	Salt Rej.	Temp Hot	Salt Conc.
-	-	Bar	LMH	%	°C	g/L
This work	PTFE+PVA1%	2.6	12.2	99.9	60	30
Fan et al. 2017 [52]	PTFE+TiO2NF	2.85	12.2	99.9	80	35
Li et al. 2020 [53]	PVDF-PTFE+PET *+CS-PEO **	0.36	19	99.95	60	20
